# Menstrual Cup Usage Among Healthcare Workers in a Tertiary Care Hospital in Ernakulam District, Kerala

**DOI:** 10.7759/cureus.79665

**Published:** 2025-02-25

**Authors:** Nileena Sunny, Vishnu B Menon, Amina Rasheed, Nandini L Rajesh, Chitra Tomy, Nikita Anil, Jeeshitha Polagani, Neha Pramodan, Megha Nair, Pavitra Sunil, Nandana Lalit, Mekha S Sreekumar, Poornasri V, Muhammad Nafiz, Nael M Iqbal

**Affiliations:** 1 Department of Community Medicine, Amrita Institute of Medical Sciences, Amrita Vishwa Vidyapeetham, Kochi, IND

**Keywords:** healthcare workers, kerala, menstrual cup, tertiary care hospital, utilization

## Abstract

Introduction

There has been a significant increase in the usage of sanitary pads, which are made of non-biodegradable plastic and harm the ecosystem. Although menstrual cups are an effective alternative, not many studies have been done to assess their usage among healthcare workers and the various factors associated with it.

Methods

A cross-sectional study was conducted among healthcare workers of the age group 18-45 years, who had collected a menstrual cup as part of the menstrual cup distribution drive in a medical college in Kerala. For the study, 103 participants were recruited after obtaining their written informed consent. Data were collected using a semi-structured questionnaire covering sociodemographic characteristics, menstrual cup usage patterns, issues faced during usage, and the reasons for not using the menstrual cup.

Results

Among the study participants, about 43% utilized the menstrual cup they received during the campaign. Comfort of using a menstrual cup was low in the first attempt but it increased with subsequent attempts. Married women had a higher chance of using a menstrual cup than unmarried women, which was statistically significant (p=0.007). Also, age was a statistically significant factor influencing menstrual cup utilization (p=0.009).

Conclusion

Even though the study was conducted among healthcare workers in a tertiary care hospital who had good awareness, menstrual cups were not used by the participants. Behavior change communication is the ideal strategy to increase the usage of sustainable methods to ensure menstrual hygiene.

## Introduction

Menstrual hygiene is one of the most underrated health concerns among the adolescent female population at present. Menstrual hygiene materials among women range from menstrual cloths, reusable/disposable pads, tampons, and menstrual cups [1]. Among these products, there is a significant increase in the usage of sanitary pads [2]. Most sanitary pads are made of non-biodegradable plastic and other harmful substances, which produce a large amount of waste that is harmful to the ecosystem [3].

An effective solution to this is the use of a menstrual cup (MC), which is a non-absorbable, bell-shaped device inserted into the vagina to collect menstrual flow [1]. The benefits of a menstrual cup are its reusability, the easy nature of its use, low expenditure, durability, and the eco-friendly nature of the product [4]. However, despite its obvious advantages, there is a low usage of menstrual cups among women of reproductive age groups. This is due to the lack of awareness, education, poor economic status, and other cultural factors [5]. Women are often faced with scrutiny and fear while exploring the use of other menstrual hygiene products. Studies about menstrual cup usage showed that there is low usage of menstrual cups among those with low awareness and low economic status [5-7]. A study conducted on the impact of imparting knowledge and awareness on the usage of menstrual cups at Alappuzha, India, reported that women who received the menstrual cups without attending the awareness session showed lower usage of menstrual cups compared to women who received awareness about menstrual cup usage, which was approximately double [8]. Another study showed that knowledge about menstrual cup usage was very low among the general population in India [9].

The Cup of Life campaign was initiated in Ernakulam to create a revolutionary change in society by creating awareness of menstruation and distributing menstrual cups. As a part of a Cup of Life drive conducted in Ernakulam, Kerala, which has been touted as the world's largest menstrual hygiene campaign, 100,000 menstrual cups were distributed within 24 hours. The campaign was initiated by the district administration in collaboration with the non-governmental organizations. The project was established with the collaboration and support of other educational institutions in the Ernakulam district. The current study was held in a tertiary care setting among medical health workers to assess the utilization of menstrual cups among women who had collected menstrual cups as a part of the Cup of Life drive and various factors such as socio-demography, comfort level of using menstrual cup, and reasons for not using the menstrual cup among non-users. The usage of menstrual cups in educated women belonging to more awareness groups, like doctors, medical students, and paramedical staff, who act as a bridge population in conveying awareness to low-awareness groups, has to be looked into.

## Materials and methods

A cross-sectional study was conducted among healthcare workers in a tertiary care hospital in Kochi from February to December 2023. The study was conducted among women of reproductive age (18-45 years), who collected the menstrual cups at the Amrita Institute of Medical Sciences, Kochi, Kerala, as part of the Cup of Life campaign. The study participants included postgraduate students, doctors, nurses, office staff and other healthcare workers of this tertiary care institution. Women who were not willing to participate and those who already attained menopause were excluded from the study.

Sample size

Based on a previous study done by Shiny Deena Varghese et al., utilization of the menstrual cup is 20.7% [8]. According to the formula, n= 4pq/d2, where p =20.7%, q= 1-p with 95% confidence interval and an absolute precision of 8%, the minimum sample size calculated was 99.

Sampling technique

The universal sampling method was used, and all the women in the reproductive age group working in the tertiary care hospital who collected the menstrual cups in the campaign were included in the study. As many as 400 participants registered for the menstrual cup drive at the hospital; however, only 142 women collected the menstrual cups. Out of them, 103 healthcare workers agreed and consented to participate in this study.

Conduct of the study

The study was done after obtaining institutional ethical committee clearance (ECASM-AIMS-2023-2024). A list of participants who had collected the menstrual cup was obtained from the administrative department. All the participants were contacted to assess their willingness and availability to participate in the study. Written informed consent was obtained from all the participants.

A pre-tested, semi-structured questionnaire was used to collect data from the study participants regarding sociodemographic details, menstrual cup usage, issues faced during the usage of menstrual cup, and the reasons for not using the menstrual cup.

Statistical analysis 

Data collected was entered into MS Excel sheet, and data analysis was done using IBM SPSS Statistics, version 21 (IBM Corp., Armonk, NY). Continuous variables were expressed as mean and standard deviation and categorical variables were expressed as frequency and percentage. The chi-square test was used to assess the association between menstrual cup utilization and factors associated with it, and a p<0.05 was considered statistically significant.

## Results

**Figure 1:**
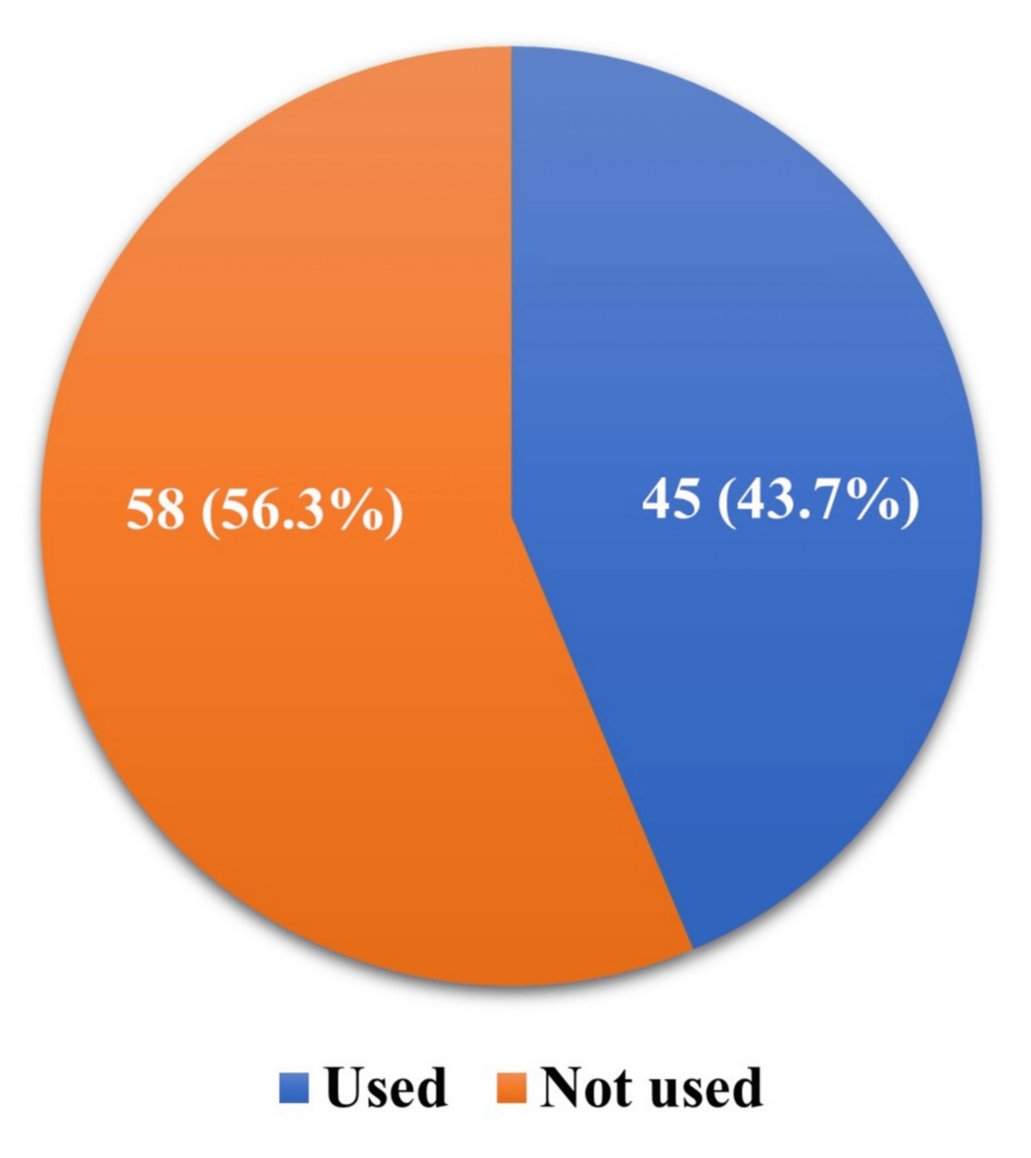
Utilization of menstrual cup among women in reproductive age group (n=103)

For the study, 103 female healthcare workers who received the menstrual cup provided their consent and participated. Regarding menstrual cup utilization, 45 (43.7%, 95% CI: 34.1-53.3%) participants used the menstrual cup they received during the awareness campaign, while 58 (56.3%) did not (Figure 1).

The mean age of the study participants was 30.4±8.3 years. More than half of the participants were married (66%, n=68), living in urban areas (60.2%, n=62), above the poverty line (76.7%, n=79), and residing with their families (81.6%, n=84). Regarding education, nearly half had completed undergraduate studies (48.5%, n=50), followed by postgraduation (37.9%, n=39), higher secondary education (8.7%, n=9), and high school education (4.9%, n=5). (Table 1)

**Table 1:** Distribution of study participants based on sociodemographic details

Sl No.	Characteristic	Category	Frequency (n= 103)	Percentage
1.	Age	18-30	54	52.4
		31-43	40	38.9
		44-55	9	8.7
2.	Marital status	Married	68	66
		Unmarried	35	34
3.	Accommodation status	Living alone	1	1
		Living with roommate	18	17.5
		Living with family	84	81.6
4.	Location	Rural	41	39.8
		Urban	62	60.2
5.	Socioeconomic status	Below poverty line (BPL)	24	23.3
		Above poverty line (APL)	79	76.7
6.	Educational status	High school	5	4.9
		Higher Secondary Schooling	9	8.7
		Undergraduate	50	48.5
		Postgraduate	39	37.9

Among those who were using menstrual cups (n=45), a majority among them (48.4%) received the information from their friends, followed by health awareness campaigns (24.3%), social media (11.7%), family (10.7%), and mass media (4.9%).

The study also evaluated participants' comfort levels with menstrual cup usage. After the first use, only nine women (20%) reported finding the menstrual cup comfortable. However, comfort levels improved significantly with repeated use, with 32 women (71.2%) feeling comfortable by the second attempt and 38 women (84.5%) by subsequent attempts. The commonly reported issues among menstrual cup users included leakage (20%, n=9), pain (20%, n=9), and discomfort during movement (17.7%, n=8). A majority of the menstrual cup users (91.1%, n=45) adhered to recommended sterilization methods, such as boiling and disinfection, and most users (82.2%, n=37) found the process easy to follow. A small proportion of users (11.2%, n=5) reported facing resistance from their family or community while discussing menstrual cup usage.

Table 2 shows the sociodemographic factors associated with menstrual cup use. Married women had a higher chance of using a menstrual cup than unmarried women, which was statistically significant (p=0.007). Additionally, a significant association was observed between age and menstrual cup usage (p=0.009). However, no significant associations were found between menstrual cup usage and factors such as location, socioeconomic status, or accommodation status of the study participants.

**Table 2:** Association between utilization of menstrual cup and associated factors.

Variable		Utilization menstrual cup (n= 103)		Chi-square value	p-value
		Used a menstrual cup (n=45)	Did not use a menstrual cup (n= 58)		
Marital status	Unmarried	9 (25.7%)	26 (74.3%)	6.96	0.007
	Married	36 (53%)	32 (47%)		
Age	18-30	17 (31.5%)	37 (68.5%)	9.41	0.009
	31-43	25 (62.5%)	15 (37.5%)		
	44-55	3 (33.33%)	6 (66.67%)		
Location	Rural	18 (44%)	23 (56%)	0.001	0.972
	Urban	27 (43.55%)	35 (56.45%)		
Socioeconomic status	Below the poverty line (BPL)	10 (41.7%)	14 (58.3%)	0.052	0.820
	Above the poverty line (APL)	35 (44.3%)	44 (55.7%)		
Accommodation Status	Living alone	1 (100%)	0 (0%)	2.16	0.280
	Living with roommate	6 (33.3%)	12 (66.7%)		
	Living with family	38 (45.2%)	46 (54.8%)		

**Figure 2:**
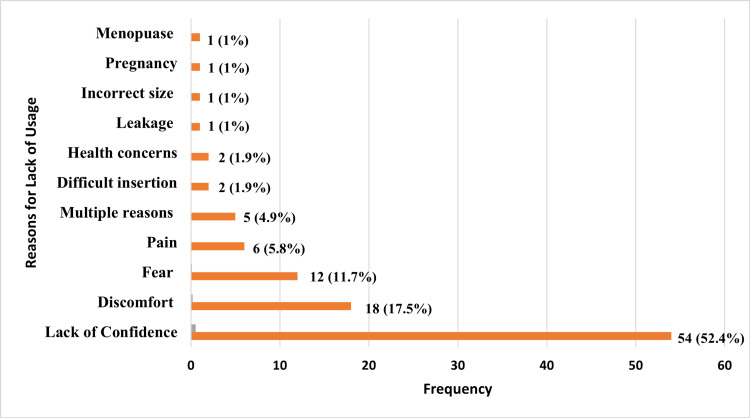
Reasons for lack of usage of menstrual cups among non-users.

On analyzing the non-users of menstrual cups (n=58), it was found that 83% (48) were aware of the economic benefits associated with using a menstrual cup. The primary reasons for not using it included lack of confidence (52.4%, n=54), discomfort (17.5%, n=18), fear (11.7%, n=12), pain (5.8%, n=6), difficulty in insertion (1.9%, n=2), and health concerns (1.9%, n=2) (Figure 2). The majority of the non-users (76%, n=58) responded that they were likely to use a menstrual cup in the future.

## Discussion

The mean age of the participants was 30.4±8.3 years, which was well within their reproductive age group. In a study done among reproductive-age women in Kerala, it was reported that among the women who received the menstrual cups without attending the awareness session, only 20.7% started using the menstrual cups, whereas 40.6% who received awareness started the usage, which is similar to our study [8]. Although our study was conducted among a more aware and educated group of the population, only 43% (45) had used the menstrual cups that they had collected from the health awareness campaign. Other studies done in Kerala reported a further decreased usage of menstrual products [10-12]. Comparatively increased usage of menstrual cups could be attributed to their accumulated life experience, which influences their preferences for eco-friendly and cost-effective menstrual solutions. A study done in Trivandrum, Kerala, found decreased usage of menstrual cups primarily due to fear resulting from insufficient awareness [6].

In our study, 66% of participants were married, and their higher openness to using menstrual cups aligns with findings by Tamilarasi et al. in Puducherry [13]. Married women tend to be more open to alternative menstrual products, whereas unmarried women may have concerns about discomfort or insertion, leading to lower adoption rates. This highlights the influence of personal experiences and marital status on menstrual product preferences.

A majority of menstrual cup users in our study were in the age group of 31-43 years, a finding consistent with research from Andhra Pradesh, where women aged 31-35 years displayed higher levels of awareness and more favorable attitudes toward menstrual cup use [14]. This could be attributed to their accumulated life experience, which influences their preferences for eco-friendly and cost-effective menstrual solutions.

Though few women were initially comfortable using menstrual cups, the comfort level increased to 84.5% with subsequent attempts. This aligns with a study by Sreedevi et al. in Kerala, where most participants found insertion and removal difficult at first attempt, but it became easier with each cycle [15]. This is due to their inexperience with menstruation cups; they will eventually acquire the proper technique for insertion and removal. It is important to educate women that the ease of use improves over time, and they should not cease using menstrual cups after their first attempt.

According to a systematic review and meta-analysis published by Anna Maria van Eijk et al., in all qualitative studies, consistent results showed that adoption of menstrual cups requires a familiarization phase over several menstrual cycles [16]. Through practice, peer support, and training, individuals become more accustomed to using the menstrual cup over time.

Among non-users, 82.8% recognized the economic benefits of menstrual cups, and 76.1% expressed willingness to use them in the future. These findings highlight a positive perception and potential for adoption, emphasizing the need for targeted interventions to address barriers and promote menstrual cups as a cost-effective menstrual hygiene solution.

Participants mainly received information about menstrual cups from friends, with minimal influence from media awareness campaigns. This reliance on informal sources may contribute to incomplete or inaccurate understanding, highlighting the need for more reliable education channels. Similar patterns have been observed in a study conducted in Ghana, indicating the influence of mass media on the adoption of reusable menstrual management materials [17].

Limitations

This study was conducted with a convenience sample among healthcare workers from a single hospital, limiting the generalizability of its findings to healthcare workers in other institutions or the general population. The small sample size further limits the internal and external validity of the results. Additionally, as a quantitative study, it did not explore the underlying reasons for the use or non-use of menstrual cups. As per extensive literature review, this is the first study done among beneficiaries of the world's largest menstrual hygiene campaign, Cup of Life, which entered the Guinness Book of World Records. The study was done among healthcare workers in a tertiary care hospital, which gave an insight into menstrual cup usage among an educated group of people in the community.

## Conclusions

Although healthcare workers in a tertiary care hospital participated in the study, there was a notable lack of usage of menstrual cups among them. As more than half of the study population was not using the menstrual cup that they collected, further behavior change communication strategies are required for increasing the utilization of menstrual hygiene materials among healthcare workers and the general population. Peer education, focusing on various aspects of cup usage such as insertion, removal, aftercare, materials, side effects, and replacement, can further alleviate fears and increase usage. Additionally, while the study found associations between menstrual cup usage with marital status and age, further qualitative research is needed to fully understand the implications of these associations.
